# Neonatal pain assessment: Do we have the right tools?

**DOI:** 10.3389/fped.2022.1022751

**Published:** 2023-02-02

**Authors:** Amelia Llerena, Krystal Tran, Danyal Choudhary, Jacqueline Hausmann, Dmitry Goldgof, Yu Sun, Stephanie M. Prescott

**Affiliations:** ^1^ Morsani College of Medicine, University of South Florida, Tampa, FL, United States; ^2^ Biobehavioral Lab, College of Nursing, University of South Florida, Tampa, FL, United States; ^3^ Department of Chemistry, College of Arts and Sciences, University of South Florida, Tampa, FL, United States; ^4^ Department of Computer Science and Engineering, University of South Florida, Tampa, FL, United States; ^5^ College of Nursing, University of South Florida, Tampa, FL, United States

**Keywords:** neonatal pain, neonatal pain assessment, neonates, clinical utility, acute pain, chronic pain, pain assessment scale

## Abstract

**Background:**

The assessment and management of neonatal pain is crucial for the development and wellbeing of vulnerable infants. Specifically, neonatal pain is associated with adverse health outcomes but is often under-identified and therefore under-treated. Neonatal stress may be misinterpreted as pain and may therefore be treated inappropriately. The assessment of neonatal pain is complicated by the non-verbal status of patients, age-dependent variation in pain responses, limited education on identifying pain in premature infants, and the clinical utility of existing tools.

**Objective:**

We review research surrounding neonatal pain assessment scales currently in use to assess neonatal pain in the neonatal intensive care unit.

**Methods:**

We performed a systematic review of original research using PRISMA guidelines for literature published between 2016 and 2021 using the key words “neonatal pain assessment” in the databases Web of Science, PubMed, and CINAHL. Fifteen articles remained after review, duplicate, irrelevant, or low-quality articles were eliminated.

**Results:**

We found research evaluating 13 neonatal pain scales. Important measurement categories include behavioral parameters, physiological parameters, continuous pain, acute pain, chronic pain, and the ability to distinguish between pain and stress. Provider education, inter-rater reliability and ease of use are important factors that contribute to an assessment tool's success. Each scale studied had strengths and limitations that aided or hindered its use for measuring neonatal pain in the neonatal intensive care unit, but no scale excelled in all areas identified as important for reliably identifying and measuring pain in this vulnerable population.

**Conclusion:**

A more comprehensive neonatal pain assessment tool and more provider education on differences in pain signals in premature neonates may be needed to increase the clinical utility of pain scales that address the different aspects of neonatal pain.

## Introduction

Comprehensive pain evaluation and management is necessary to support the wellbeing of vulnerable newborn infants (1). Despite evidence showing that neonatal pain is associated with both short term and long-term adverse outcomes, there are insufficient means to adequately assess neonatal pain (2–4). Specific areas of concern when neonates experience frequent or uncontrolled pain include altered brain development, neurodevelopment, pain perception, and poor regulation of stress (5–7). As infants are unable to verbally self-report pain, they are fully dependent on care providers to accurately assess and respond to their pain (4). Preterm infants are at increased risk for undertreated pain as they are more likely to undergo a greater number of painful procedures as compared to full term infants (2). Additionally, the Epidemiology of Procedural PAin In Neonates (EPIPPAIN) study, demonstrated that neonates experienced a median of 10 painful procedures daily during their NICU hospitalization, yet only 20% of procedures were accompanied by analgesic treatment (8).

Parents and healthcare providers indicate that the assessment of pain is a major concern in the care of neonates (6). Additionally, healthcare providers indicate insufficient education on and lack of validated scales to properly evaluate neonatal pain (9). In spite of recognized need for more comprehensive evaluation, a gold standard means of neonatal pain assessment has yet to be selected due to the limited reliability, validity, and clinical utility of existing tools (10). Further, there is distinct variation among how the currently available assessment tools measure pain and which factors are included in the assessment. Many scales include only physiologic measures that can be objectively computed such as respiration rate, while others only include behavioral measures as physiologic measures may be impacted by stress or other conditions (6, 10). Other scales include caregiver perceptions and contextual measures such as gestational age or change in activity (11). The challenge for consistency increases when pain measures are subjective in nature (10). Inconsistent correlations between behavioral, physiological, and cortical measures of pain complicate the selection of scales to comprehensively evaluate neonatal pain (11, 12). The lack of consensus regarding which items to include in a scale to evaluate neonatal pain hinders comparison among all the published assessment tools (10).

There are several barriers to the creation and implementation of a comprehensive neonatal pain assessment tool including lack of provider education on pain assessment, impracticality or invalidity of currently available pain assessment instruments, and variation in pain response among infants (10). One barrier to the development of a standardized pain assessment tool is age-dependent variation in pain response. For example, preterm infants may lack the motor skills to express pain compared to a full-term infant (1, 7, 13). Another barrier to the standardization of pain assessment tools is the non-specific nature of pain symptoms, for example crying can indicate hunger, the need to be held, agitation, or a soiled diaper in addition to pain (4). The inability for neonates to adequately express pain coupled with caregivers' lack of ability to interpret neonatal pain signals emphasizes the need for multiple dimensions of pain assessment in neonates. Further, clinical utility is a concern. The lack of a valid and reliable instrument to serve as a gold standard for assessing neonatal pain not only hinders the ability to diagnose and treat neonatal pain, but also increases the time and cost of educating caregivers on multiple assessment measures that inadequately perform the desired function(4, 10).

In this review, we did not seek to comprehensively evaluate all known pain assessment scales with meta-analysis of statistical validation as Giordano et al., did in 2019 (14). Instead we sought to determine which pain assessment tools are most commonly being used for neonates, consolidate the available research, and extract relevant issues that continue to hinder pain scale development and use in this population. Several themes arose in the literature that we reviewed including indications for tool use and pain intervention, provider perceptions, ability of extreme preterm neonates to localize and display pain, and family involvement. We compared and consolidated the research and extracted the themes addressed to highlight the strengths and limitations of currently available neonatal pain assessment scales. There is a need for a comprehensive and standardized measurement of neonatal pain to alleviate suffering and promote the wellbeing of vulnerable infants (4). In this study, we assess and compare original research on currently available pain assessment tools used to evaluate neonatal pain in the clinical setting.

## Methods

### Study selection and characteristics

**Figure 1:**
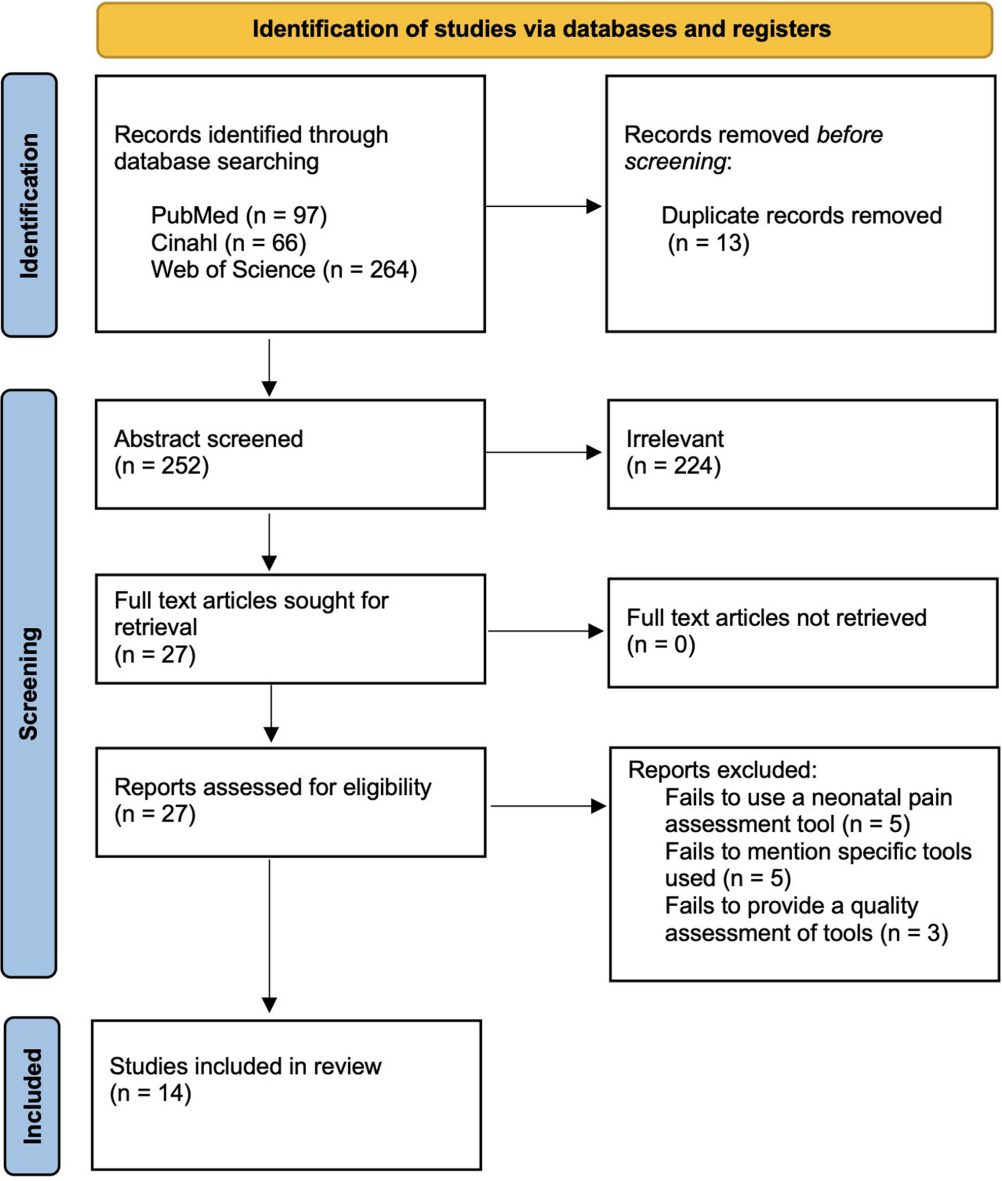
PRISMA flow diagram.

We searched the literature using the key words “neonatal pain assessment” through Web of Science, PubMed, and CINAHL. All selected studies are in English, original research, and published between 2016 and 2021. Duplicates were deleted. After initial screening of the abstracts, we based inclusion criteria on relevance to neonatal pain assessment and evaluation of the effectiveness of the pain assessment instruments. After full article review, we excluded studies that failed to use or mention specific pain assessment instruments, or those that failed to provide a quality assessment of the chosen pain scale. We selected studies that evaluate pain measured during the first 28 days of life and reviewed the research for each instrument's reported ability to measure acute, chronic, and continuous neonatal pain. Further, we evaluated each pain assessment tool's reported clinical utility, inter-rater reliability, family perception, and provider insights. Three authors independently screened the abstracts, while the third author independently settled disputes. We subsequently added 1 article that was not selected using our search criteria but was recommended by independent reviewers prior to publication. Summary data of the included studies are included in Figure 1.

We grouped common themes that emerged from the studies evaluating neonatal pain assessment scales into eight categories: measurement of behavioral parameters, physiological parameters, continuous pain assessment, acute pain measurement, chronic pain measurement, high inter-rater reliability, ability to distinguish between pain and stress, and clinician's usability rating. Additionally, we studied common themes impacting the assessment of neonatal pain among the chosen articles including policy guidelines, family perceptions, nurse attitudes and education, and specific indications of neonatal pain.

### Eligibility & exclusion criteria

Our initial search results yielded 265 studies. After removing 13 duplicate studies, we excluded 225 studies for failing to assess pain assessment tools, not pertaining to neonatal pain assessment, or relating to nonhuman species. After full review of the remaining 27 studies, we excluded 12 additional articles for irrelevance or a poor-quality rating utilizing the NIH Quality Assessment Tool for Observational and Cohort and Cross-Sectional studies, leaving 14 studies that scored either good or fair to be included in the review (Table 1). One study that was published in 2022 was subsequently added. Studies were scored by three independent reviewers, with any disagreements assessed by a fourth independent reviewer.

**Table 1:** Qualitative assessment of observation and cohort and cross-sectional studies involving neonatal pain assessment tools.

Article	Assessment Scale Evaluated	Type of Study	Rating* (good/fair/poor)
Taplak et al. (2019)	PIPP-R	Observational Validation Study	Good
O'Sullivan et al. (2016)	PAT and COVERS	Observational Cohort Study	Good
Kappesser et al. (2019)	NFCS-R, PIPP-R, NIPS, N-PASS, BPSN	Observational Validation Study	Good
Raffaeli et al. (2017)	EDIN6, EDIN	Observational Cohort Study	Fair
DiLorenzo et al. (2017)	NFCS, MBPS	Longitudinal Cohort Study	Fair
Walas et al. (2020)	NIPE	Observational Cohort Study	Good
Cremillieux et al. (2018)	NIPE	Observational Validation Study	Good
Cignacco et al. (2017)	BPSN	Observational Validation Study	Good
Huang et al. (2018)	PIPP-R, N-Pass, NIAPAS	Observational Validation Study	Good
Orovec et al. (2018)	PIPP	Observational Cohort Study	Fair
Ilhan et al. (2021)	PAT	Observational Validation Study	Fair
Desai et al. (2018)	N-PASS and NIPS	Observational Validation Study	Good
Fortney et al. (2020)	COMFORT-B, NIAPAS	Observational Validation Study	Good
Walas et al. (2022)	NIPE	Observational Cohort Study	Good

* Ratings derived from NIH Qualitative Assessment of Observational and Cohort and Cross-Section Studies. A score of ≥11/14 = good, 7–10/14 = fair, <7/14 = poor.

## Results

The identified studies assess several internationally used, pain assessment instruments including ALPS-Neo (3), BPSN (10, 11),CHIPPS (10), COMFORT-B/neo (1), COVERS (15), EDIN (3)/EDIN6 (7), NFCS-R (10), NIAPAS (16), NIPE (17, 18), NIPS (4, 10), N-PASS (1, 4, 10, 16), PAT (13 15), and PIPP-R (2, 10, 16, 19). The tools assessed had varying results in correspondence to the eight categories upon which they were evaluated (Table 2).

**Table 2:** Neonatal Pain Assessment Tool Comparison.

Neonatal Pain Assessment Tool	Full Name	Behavioral Parameters	Physiological Parameters	Continuous Pain Assessment	Acute Pain Measurement	Chronic Pain Measurement	High Inter-rater Reliability	Distinguish Between Pain and Stress	High Usability
COMFORTneo	Comfort Assessment, Neo Scale	+	X	+	+	+	X	+	O
ALPS-Neo	Astrid Lindgren Children's Hospital Pain Scale	+	X	+	O	O	O	O	O
N-PASS	Neonatal Pain, Agitation and Sedation Scale	+	+	+	+	+	+	+	O
COMFORT-B	COMFORT Behavior Scale	+	+	O	O	O	O	O	X
NFCS-R	Neonatal Facial Coding System	+	X	O	+	O	+	+	X
CHIPP's	Children and Infants Postoperative Pain Scale	+	X	O	+	O	+	+	X
EDIN	Échelle de Douleur et d'Inconfort du Nouveau-né	+	X	+	O	X	O	O	O
BPSN	Bernard Pain Scale for Neonates	+	+	O	O	O	O	+	O
NIPS	Neonatal Infant Pain Scale	+	X	X	+	X	O	+	O
NIPE	Newborn Infant Parasympathetic Evaluation	X	+	+	X	O	O	O	O
PIPP-R	Premature Infant Pain Profile-Revised	+	+	O	+	+	X	+	+
NIAPAS	Neonatal Infant Acute Pain Assessment Scale	+	+	O	+	O	+	+	X
PAT	Pain Assessment Tool	+	+	O	+	O	+	O	+
EDIN6	Échelle de Douleur et d'Inconfort du Nouveau-né (Includes a factor for Postmenstrual Age)	+	+	+	O	X	O	O	O
COVERS	The COVERS Scale	+	+	O	+	O	+	O	O
(+) Factor Included	(X) Factor Excluded	(O) Further Investigation Needed							

ALPS-Neo is an adaptation of the Astrid Lindgren Children's Hospital Pain Scale (ALPS-1) pain assessment scale designed for term newborns. Both scales are based on 5 behavioral observations including facial expression, breathing pattern, tone, hand/foot activity, and level of activity, but the ALPS-Neo is adapted to include premature infant behaviors (20). ALPS-Neo had high inter-rater reliability and usability and was proficient in identifying continuous pain, however it lacked physiologic parameters and further investigation is needed to determine if it may distinguish between pain and stress (3).

Bernese Pain Scale for Neonates (BPSN) is a multidimensional pain scale, meaning that it includes both behavioral and physiologic (heart rate and oxygen saturation) measures. It is valid in term and preterm neonates above 27 weeks receiving respiratory support (21). It distinguished distress and pain in certain neonatal populations but had low inter-rater reliability possibly as a result of individual contextual factors not addressed in the scale that influenced pain responses (11).

Children and infants post-operative pain scale (CHIPPS) is a unidimensional pain scale measuring behavioral indicators of post operative pain in term and preterm infants. It was proficient in measuring acute pain and distinguished situations characterized by distress or pain. It had high inter-rater reliability. CHIPPS provided no manuals or training, limiting its usability and functionality, and failed to account for physiological parameters (10).

COMFORT-B is a revision of the COMFORT scale that was derived for PICU patients and postoperative pain in children (22). The COMFORT B scale removes the physiologic measures that were included in the original COMFORT scale that included both behavioral and physiologic measures. It proved proficiency in behavioral and physiological parameters yet lacked usability and functionality and had moderate interrater reliability (1). COMFORTneo is a revision of the COMFORT scale adapted for preterm neonates. It measures unidimensional behavioral assessments and was proficient in testing continuous pain, but lacked high inter-rater reliability, though there was a clinical guideline for daily operation (23).

COVERS (Crying, Oxygen requirement, Vital signs, Expression, Resting, and Signaling distress) contains both behavioral and physiologic measures, and has good clinical utility, but is not validated in sedated, paralyzed, or extremely preterm infants (15, 24).

Échelle de Douleur et d'Inconfort du Nouveau-né (EDIN) is a unidimensional behavior-based pain scale. (EDIN6) is a modification to the EDIN scale that includes post menstrual gestational age to make the scale more valid for preterm infants (7). The researchers found that the scales were proficient in testing continuous pain (3, 7). The scales lacked the ability to assess chronic pain measurements, however, and required further investigation for its inter-rater reliability (7).

Neonatal Facial Coding System (NFCS/NFCS-R) measure of infant pain-related distress in known pain-specific contexts using 10/5 individual facial actions. Scales distinguished procedural distress and pain and had high inter-rater reliability but lack usability because of strenuous and time-consuming training. They are not designed to measure chronic pain or stress. They do not account for physiologic parameters (10).

Neonatal Infant Acute Pain Assessment Scale (NIAPAS) is a multidimensional measure measuring both behavioral and physiologic markers of pain. It distinguished situations characterized by distress or pain, had high usability, but lacked high inter-rater reliability (16).

Newborn Infant Parasympathetic Evaluation Index (NIPE) measures short term heart rate variability surrounding painful procedures and is very proficient in assessing physiological parameters but lacked the ability to assess chronic pain or acute pain independent of known painful procedures (17, 18). It does not require human scoring.

Neonatal Infant Pain Scale (NIPS) is a unidimensional scale measuring 6 behavioral parameters. This scale distinguished distress or pain and measured acute pain but failed to assess chronic or continuous pain (4, 10).

Neonatal Pain Agitation and Sedation Scale (N-PASS) is a multidimensional scale that scores behavioral and physiologic parameters for both pain and sedation. NPASS was proficient in assessing continuous, acute, and chronic pain, and was able to distinguish distress and pain in neonates of all gestational ages. It had high inter-rater reliability when scorers received adequate training. In clinical practice, training was often insufficient leading to inter-rater inconsistencies that limited the utility of this tool (1, 4, 16).

Pain Assessment Tool (PAT) is a multidimensional scale containing physiologic, behavioral, and nurse perception scores. It has been adapted for the neonatal population by additional descriptions to aid provider scoring. It was able to distinguish between pain and distress, had good interrater reliability, but had poor clinical utility compared to other scales (15).

Premature Infant Pain Profile (PIPP) is a multidimensional pain scale that includes measures of behavioral, physiologic, and contextual factors. PIPP-R is revised by scoring the contextual factors (gestational age and behavioral state) only if there is a change in behavioral and physiologic factors after the painful event (12). Researchers found that the PIPP-R was proficient in measuring behavioral and physiological parameters, distinguished distress and pain, and could measure continuous, chronic, and acute pain, but lacked inter-rater reliability and presumed that because low gestational age infants could not express pain, that they did not feel pain (13, 16).

Many of the selected studies compared the different pain assessment tools to one another. In a study comparing N-PASS and NIPS, N-PASS generated 98% of scores greater than yielded by NIPS (4). N-PASS showed significant distinction in assessing acute and chronic pain in comparison to NIPS (4). Another study compared the usage of EDIN and EDIN6, where it was determined that EDIN6 pain assessment readings were 3 times greater than that of EDIN (7). One study evaluated the effectiveness of PIPP-R, N-PASS, and NIAPAS in a 60-bed NICU in China (13) and reported that all three scales were correlated with one another, with 55.9% of nurses giving preference to N-PASS, and 23.5% and 20.6% to NIAPAS and PIPP-R respectively, making N-PASS the most feasible and functional amongst the three pain assessment tools evaluated (13).

## Discussion

In our systematic review, we assessed the research evaluating 13 neonatal pain scales currently in use to measure neonatal pain. The researchers in these studies identified strengths and limitations in all tested pain scales that impacted their use for measuring neonatal pain in the clinical setting. No scale excelled in all areas identified as important for reliably recognizing and measuring pain in this vulnerable population. In reviewing the research surrounding these pain scales, eight categories were identified in the different scales and literature as important indicators for adequately assessing neonatal pain.

1. Behavioral expressions of pain such as facial expressions, body movements, changes in tone, changes in vocalization, changes in level of consciousness, and attention changes (25).
2. Physiological manifestations of pain including changes in heart rate, blood pressure, withdrawal reflex, respiratory rate, oxygen saturation, skin color (26).
3. Contextual contributors to pain such as gestational age, postmenstrual age, and critical illness that can contribute to alterations in pain expression (13).
4. Identify acute-transient, acute continuous, and chronic pain (3)
5. Distinguish between pain and stress (18).
6. Provider and parent input
7. High inter-rater reliability
8. Clinical utility (17).

Though pain assessment is considered an important vital sign, standardized pain assessment scales are not always available for use at the bedside, and many nurses report difficulty assessing pain without them (27, 28). The researchers in these studies found clinical utility to be an important factor that contributes to an assessment tool's success. This assertion is supported by Manworren and Stinson who noted that different strategies for pain assessment alter the clinical usability of a tool and that variability in pain scores has significant clinical impact (29). In Popowicz et al., medical personnel in the NICU identify additional barriers to clinical integration to include insufficient training, haste, and unwillingness to change current practices (30). Additionally, some infants such as premature infants and infants with decreased levels of consciousness may be unable to localize and exhibit pain as healthy term infants are able to do, thus pain scales must be able to account for pain in these populations and providers must be educated to recognize pain in these infants (31, 32). Overall, our review reveals the complexity of neonatal pain assessment and identifies critical areas for improvement among pain assessment tools.

In our review of the literature, physiological measures were utilized in less than half of the pain assessment scales, while behavioral parameters were most consistently utilized. Physiologic measures of pain such as heart rate variability, respiratory rate variability, oxygen saturation changes, and blood pressure changes that are conveniently measurable by bedside providers do not always correlate with other measures of pain because they are influenced by contextual factors such as level of consciousness, prematurity, medication, anxiety, stress, fear, and temperature. Physiological parameters alone may add ambiguity to the assessment of pain; for example, increased heart rate may indicate stress or excitement in addition to pain (33, 34). Unfortunately, behavioral measures suffer from the same shortcomings. Behavioral measures when used in isolation are subject to bias and inter-rater variation (35). Age-dependent variation may further complicate the assessment of preterm pain assessment as preterm infants may lack the motor skills or behavioral range to express pain compared to a full-term infant (1, 7, 13).

Walas et al. pilot the integration of multi-dimensional physiologic measures using NIPE and skin conductance activity following a heel stick procedure to assess parasympathetic and sympathetic nervous system tone changes, respectively. Physiologic changes, for example heart rate variability and skin conductance, may signal acute changes in neonatal condition; their addition to behavioral pain assessment tools may increase pain assessment scale reliability (18). The use of behavioral and physiological parameters together may improve neonatal pain assessment, but may also be insufficient in certain circumstances (33). For example, the use of both measures in the COMFORT tool allowed for the differentiation of pain intensities before, during, and after a procedure according to a study by Franck et al. (35), but behavioral and physiological measures do not always correlate, and contextual parameters, such as temporality of pain or age of neonate, can explain discrepancies (13, 34, 36). Increased integration of multidimensional data may aid in the assessment and management of neonatal pain, but the difficulty lies in the lack of a gold standard upon which to compare and validate pain assessment tools. In adults, the gold standard is self-report, though this measure is unavailable in the pre-verbal infant complicating the development of pain assessment scales in this population (37).

Acute pain is the most assessed outcome among the assessment tools, yet preterm infants, for whom pain localization and expression is suppressed or delayed, are likely to have a prolonged stay in the NICU in which they undergo prolonged period of frequent painful interventions (3). In the setting of intubation, or with handling during incomplete skin keratinization, extreme preterm infants may experience pain but be unable to localize or express it. Chronic pain is problematic to measure in these circumstances, and traditional definitions of pain chronicity may not apply to infants as they have not lived long enough to have meet criteria for chronic pain applied to older children and adults (36). Inability to predict or adequately measure chronic pain complicates experimental design, so most studies are designed to measure acute pain in predictable situations such as heel lance or during procedures. Persistent pain has been shown to cause long-term effects on childhood development through alterations in somatosensory processing and injury-induced neural plasticity (5, 6). Of the studies identified in this review, only the COMFORTneo and N-PASS differentiate pain stage and assess pain temporality (4, 23). Activity level, facial expression of pain (i.e., grimaces), and poor response to handling may indicate chronic pain in the preterm population (36). Developing tools that identify and measure both acute and chronic pain is imperative to reduce suffering and prevent pain-related neurodevelopmental outcomes in neonatal and preterm infants.

A pain assessment scale's ability to differentiate between pain and stress may allow early interventions that prevent extensive adverse effects associated with chronic pain. Behavioral parameters may contribute to the differentiation between stress and acute pain (10). PIPP-R, NIPS, NFCS, N-PASS and BPSN can all differentiate between stress and acute pain indicated by significantly higher scores during painful procedures but are less valid in the setting of chronic or persistent pain particularly in very preterm infants. Behavioral and physiologic signs of distress may predict pain progression; stress and pain may represent a continuum rather than separate entities. Identification of stress before pain may allow for increased utilization of non-opiate pain interventions such as sucrose administration prior to procedures and parental touch (38, 39). Tools that can discriminate between a stressful event and a painful event may enhance the management of pain in neonates (15). The difficulty lies in validating such measures when the infant is unable to localize or express pain in a manner that can be recognized.

Aside from difficulties in experimental design to recognize and assess chronic or persistent pain, or the pain of preterm and very preterm infants, there were areas of potential bias in the studies selected. Unblinded outcome assessors and memory biases due to delayed data collection impacted some of the selected studies. Studies that assessed for bias often did so by incorporating blinds for the data collectors (10, 19, 40). The methodology of our systematic review may be subject to selection bias. One potential example of bias is the inclusion of studies printed in English only. Additionally, we did not search by the names of known assessment tools as we wanted to capture the scales most currently evaluated. Our review provides specific examination into the current measures used to evaluate pain in neonates We identify strengths of each assessment scale and areas for improvement, that if addressed, will ultimately improve the clinician's ability to detect and respond to neonatal pain.

A more comprehensive neonatal pain assessment tool may be needed to increase clinical utilization while addressing the complexity of neonatal pain. The ideal tool will incorporate behavioral as well as physiological parameters, detect early signs of pain and distinguish pain from stress. It will include contextual factors that influence the expression of pain such as gestational age and level of consciousness. It will include provider and parental input and identify the early stages of pain, in addition to having a high clinical usability and inter-rater reliability. Of the 13 scales we study, none comprehensively addresses all areas needed to reliably identify and manage neonatal pain in all contexts. Overall, the N-PASS tool scored the best on the criteria we selected compared to the other tools. Although it scores well, N-PASS has high inter-rater variability in clinical practice as raters often receive little to no training (1). Inadequate training and support were frequently noted in studies assessing medical provider perceptions and clinical behaviors (30, 39). In their systematic review, Popowicz et al. explores this phenomenon; noting the importance of extensive education as a method for increasing clinical awareness and integration (41). Further tool development may be necessary to tackle the limitations of existing tools and promote the wellbeing of this vulnerable population (15).

While the development of an ideal tool for measuring all aspects of neonatal pain including chonic and acute pain in addition to the ability to distinguish stress may seem unfeasible within the context of our current human limitations, continuous monitoring and predictive algorhythms develoved by artificial intelligence (AI) may provide an answer. Using objective variables such as heart rate and facial movements, AI frameworks could be used to make high-confidence predictions to identify a therapeutic window before pain onset, thus extending the bedside provider's ability to detect pain without the need for extensive re-education and training. Continuous AI monitoring supports proactive use of safer, non-narcotic pain interventions that reduce neonatal suffering and avoid opioid withdrawal (42). Although AI is one potential avenue for improved pain management it has yet to be fully studied. There may be other options including individual scales for different types of neonatal pain or distress, or point of care testing of physiologic markers of pain or distress. Improvements in neonatal pain management may require such creative solutions in addition to well validated and reliable assessment tools to comprehensively assess all types of neonatal pain at multiple gestational ages, and well-designed trials that identify biomarkers or classifiers of neonatal pain for use as a gold standard for validity testing, especially in the context of prematurity and chronic pain.

## Data Availability

The original contributions presented in the study are included in the article/Supplementary Material, further inquiries can be directed to the corresponding author.
